# Activating Transcription Factor-5 Knockdown Reduces Aggressiveness of Mammary Tumor Cells and Attenuates Mammary Tumor Growth

**DOI:** 10.3389/fendo.2017.00173

**Published:** 2017-07-21

**Authors:** Sarit Ben-Shmuel, Rola Rashed, Ran Rostoker, Elina Isakov, Zila Shen-Orr, Derek LeRoith

**Affiliations:** ^1^Clinical Research Institute at Rambam (CRIR), Diabetes and Metabolism Clinical Research Center of Excellence, Rambam Medical Center, Haifa, Israel; ^2^The Ruth and Bruce Rappaport Faculty of Medicine, Technion – Israel Institute of Technology, Haifa, Israel; ^3^Division of Endocrinology, Diabetes and Bone Diseases, Icahn School of Medicine at Mount Sinai, New York, NY, United States

**Keywords:** breast cancer, activating transcription factor-5, CD24, autophagy, insulin

## Abstract

Activating transcription factor-5 (ATF5) is an anti-apoptotic factor and has been implicated in enhancing the survival of cancer cells under stress and in regulating the autophagy process. Targeting ATF5 in anticancer therapy may be particularly attractive because of its differential role in cancer cells than in non-transformed cells, thus allowing specificity of the treatment. Using the delivery of short hairpin RNA vectors into the Mvt1 and Met1 cell lines, we tested the role of ATF5 in the development of mammary tumors *in vivo* and in regulating proliferation and migration of these cells *in vitro*. In this study, we demonstrate that knockdown of ATF5 (ATF5-KD) in both cell lines results in a decreased tumor volume and weight, as well as in a reduced proliferation rate and migratory potential of the cells. In addition, ATF5-KD led to an increased autophagy flux and a shift in the sub-populations comprising Mvt1 cells from the aggressive CD24-positive cells toward less aggressive CD24-negative cells. Taken together, these findings suggest that ATF5 plays an important role in enhancing mammary tumor cells overall aggressiveness and in promoting mammary tumor growth and emphasize the possible benefit of anti-ATF5 therapy in breast cancer patients, particularly, against tumors characterized with the positive expression of cell surface CD24.

## Introduction

The association between type 2 diabetes (T2D) and an increased risk of breast cancer incidence and mortality has been confirmed in numerous studies and meta-analyses (1–3). The key features of T2D, namely, hyperglycemia, insulin resistance, and hyperinsulinemia, as well as increased bioavailability of insulin-like growth factor-I (IGF-I), have been indicated as potential molecular mechanisms underlying this association (4–6). Specifically, the mammary tumor effect of hyperinsulinemia was demonstrated in females of the transgenic MKR mouse model of T2D, inoculated with the mammary cancer cell lines, Met1 (7) and Mvt1 (8), where tumors derived from both cell lines showed a significant increase in growth rate in MKR females compared with wild-type mice.

It was demonstrated that the mitogenic effects of both insulin and IGF-I are mediated, among others, through the phosphatidylinositol 3-kinase (PI3K)–Akt pathway (9), which is known to play a significant role in cell proliferation, survival, and migration, thereby promoting tumor growth and invasiveness (10, 11). Recently, we have shown that insulin or IGF-I stimulation of Mvt1 cells leads to the upregulation of several mRNA transcripts, associated with cell proliferation and migration, such as Cyclin D1 and the transcription factor ETS2, as well as to the downregulation of transcriptional repressor, high mobility group (HMG) box-containing protein 1 (HBP1) (12). At the same time, we demonstrated that knockdown of insulin receptor in Mvt1 cells is associated with the downregulation of CD24-expressing cells. We have shown that Mvt1 expressing CD24 on their cell surface (CD24-positive cells) are more aggressive than Mvt1 cells not expressing CD24 on their cell surface (CD24-negative cells) and that inoculation of CD24-expressing cells into the mammary fat pad of female mice results in significantly larger tumors compared to inoculation with CD24-negative cells (12, 13). Interestingly, gene expression analysis between CD24-positive and -negative Mvt1 cells revealed activating transcription factor-5 (ATF5) as one of the top 10 genes that were upregulated in the aggressive CD24-positive cells over CD24-negative cells (13).

Activating transcription factor-5 (also known as ATFx) is a member of the ATF/cAMP response element-binding family of transcription factors (14). This transcription factor is an anti-apoptotic factor (15) and has been implicated in enhancing the survival of cancer cells under stress, such as serum deprivation, chemotherapy, and irradiation (16–18). In addition, ATF5 plays a role in the regulation of autophagy (19). During autophagy, intracellular components are engulfed by unique double-membrane autophagosomes, which during their formation, microtubule-associated protein 1 light chain 3 (LC3) in its cytosolic form (LC3-I) is conjugated with phosphatidylethanolamine to LC3-II (20). Later on, p62 (also known as SQSTM1) is selectively incorporated into the completed autophagosome by directly binding to LC3 and is subsequently degraded in the autolysosome (21). It was shown that knockdown of ATF5 (ATF5-KD) in transformed murine myeloid cells increased the protein levels of LC3-II while p62 levels were decreased, indicating the successful formation of autophagolysosmes and overall induction of autophagy (19). Interestingly, the attractiveness of ATF5 gene targeting as an anticancer therapy also emanates from its differential role in cancer cells than in non-transformed cells. Several reports show that ATF5 mediates the survival of breast cancer, glioma, and transformed myeloid cells but is not required for the survival of non-transformed astrocytes, fibroblasts, and breast epithelial cells (16, 19, 22), thus enabling specificity of ATF5 clinical targeting.

In light of the important role of ATF5 in the regulation of proliferation, survival, migration, and overall aggressiveness of cancer cells, we sought to investigate the effects of ATF5 knockdown in Mvt1 and Met1 murine mammary tumor cell lines.

## Materials and Methods

### Animals

FVB/N mice were used in this study. Mice were fed standard chow and water *ad libitum* and were kept on a 12 h light/dark cycle. This study was carried out in accordance with the recommendations of the National Institutes of Health Guide. The protocol was approved by the Technion Animal Inspection Committee.

### Cell Culture

Mvt1 mouse mammary cancer cells were derived from MMTV-c-Myc/VEGF transgenic FVB/N mice tumor explants, as previously described (23). Met1 cells were derived from MMTV-PyVmT transgenic FVB/N mice (24). Cells were cultured in Dulbecco’s modified Eagle’s medium (Biological Industries, Beit Haemek, Israel), supplemented with 10% fetal bovine serum (FBS, Biological Industries) and 1% penicillin:streptomycin (Biological Industries). Cells were maintained at 37°C in a humidified atmosphere consisting of 5% CO_2_ and 95% air.

### ATF5-KD by Lentiviral-Based Delivery of Short Hairpin RNA (shRNA)

Vectors encoding shRNA-directed against mouse ATF5 (Genecopoeia, Rockville, MD, USA) were transfected into 293FT cells, along with ViraPower Lentiviral Packaging Mix (ThermoFisher Scientific, Waltham, MA, USA), using Lipofectamine^®^ 2000 Transfection Reagent (ThermoFisher Scientific) according to the manufacturer’s recommendations. Medium was refreshed after 18 h. Virus-containing medium was collected after additional 24 h, filtered through 0.45 µm Filter Unit (Merck Millipore, Billerica, MA, USA), and placed on adherent Mvt1 or Met1 cells in the presence of 8 µg/ml polybrene (Sigma-Aldrich, St. Louis, MO, USA). A stable ATF5-KD was achieved by selection of the infected Mvt1 and Met1 cells (obtained from a pool of clones) with 2 µg/ml puromycin (Sigma-Aldrich). Cells infected with a vector containing a scrambled shRNA sequence (pool of clones) were used as control cells (scrambled).

### Experimental Designs

For insulin and IGF-I treatment experiments, Mvt1 cells were seeded onto six-well plates at a concentration of 3 × 10^5^ cells/well and allowed to settle for 24 h. Cells were then starved with serum-free medium (SFM) containing 1% bovine serum albumin (BSA) overnight. Cells were treated with wortmannin (PI3K inhibitor, 100 nM; Sigma-Aldrich) or vehicle for 30 min before the addition of 10 nM of insulin (Actrapid^®^; Novo Nordisk) or IGF-I (R&D Systems, Minneapolis, MN, USA) for 4 h.

For cleaved caspase 3 and autophagy marker expression analysis, Mvt1 or Met1 scrambled and ATF5-KD were seeded onto six-well plates at a concentration of 3 × 10^5^ cells/well and allowed to settle for 24 h. Cells were then starved with SFM + 1% BSA for at least 72 h to induce autophagy.

### Real-time PCR

Total RNA was extracted using Total RNA Purification Kit (Norgen Biotek Corp., Thorold, ON, Canada) and reversed transcribed into cDNA using Verso cDNA Synthesis Kit (ThermoFisher Scientific). Real-time PCR was performed on a Rotor-Gene 6000 (Qiagen, Hilden, Germany) or Eco Real-time PCR System (Illumina, San Diego, CA, USA), using Absolute Blue QPCR Mix (ThermoFisher Scientific) according to the manufacturer’s recommendations. Amplification specificity was verified by melting curve analysis. Values of mRNA expression were normalized to the level of B2M expression. The oligonucleotide primers used were as follows:

ATF5 sense 5′-AATTGAGGTGTATAAGGCCCG-3′
ATF5 antisense 5′-GGATAGGAAAGTGGAATGGAGG-3′
B2M sense 5′-TTCTGGTGCTTGTCTCACTGA-3′
B2M antisense 5′-CAGTATGTTCGGCTTCCCATTC-3′

### Syngeneic Orthotopic Tumor Models

Mvt1-scrambled and ATF5-KD cells were detached with trypsin solution (Biological Industries) into single cells and suspended in phosphate-buffer saline (PBS) at a concentration of 0.5 × 10^6^ cells/ml. A total of 100 µl (50,000 cells) were then injected into the left inguinal mammary fat pads (no. 4) of FVB/N females at 7–9 weeks of age.

Met1 scrambled and ATF5-KD cells were similarly inoculated into the mammary fat pads of FVB/N females (500,000 cells per mouse).

Tumor volume was monitored once a week using calipers and calculated in cubic millimeter by the formula: width^2^ × length × 0.5. At sacrifice, tumors were removed and weighed.

### Proliferation Assay

Mvt1 or Met1 scrambled and ATF5-KD cells were seeded onto 96-well plates at a concentration of 500 cells/well and were grown for 72 h. Proliferation assay was performed at times 0 and 72 h using CyQUANT^®^ NF Cell Proliferation Assay Kit (ThermoFisher Scientific), according to the manufacturer’s instructions. Proliferation fold was calculated as the ratio between the fluorescent values at the end of the experiment and time 0.

### Western Blot Analysis

Mvt1 or Met1 scrambled and ATF5-KD cells were lysed in lysis buffer (10 mM Tris–HCl, pH 7.5, 150 mM NaCl, 10 mM sodium pyrophosphate, 1 mM β-glycerolphosphate, 1 mM Na_3_VO_4_, 50 mM NaF, 1.25% CHAPS (3-[(3-cholamidopropyl)dimethylammonio]-1-propanesulfonate), and Complete^®^ Protease Inhibitor Cocktail (Roche Diagnostics, Basel, Switzerland)). Lysates were rotated on ice for 30 min and centrifuged at 13,000 rpm for 10 min. Supernatants were collected, and protein concentrations were determined with Protein Assay Kit (Bio-Rad, Richmond, CA, USA). Protein (20 µg for Akt activation analysis or 40–45 µg for cleaved caspase 3 and autophagy markers) was electrophoresed through 10 or 12% polyacrylamide gel and transferred to a nitrocellulose membrane. The membranes were immunoblotted with the desired antibody, followed by an appropriate secondary antibody conjugated with horseradish peroxidase (Jackson ImmunoResearch Laboratories, West Grove, PA, USA). Immunoreactivity was detected by an enhanced chemiluminescence (WesternBrightTM Quantum Western blotting detection kit, Advansta, Melano Park, CA, USA), using luminescent image analyzer LAS-4000 (Fujifilm, Tokyo, Japan). Densitometry analysis was performed using the ImageQuant software (GE Healthcare Bio-Sciences, Pittsburgh, PA, USA).

The following antibodies were used: Phospho-Akt (Thr308), total-Akt, caspase 3, SQSTM1/p62, and β-actin from Cell Signaling Technology (Danvers, MA, USA); ATF5 (clone EPR18286) from Abcam (Cambridge, UK); and LC3 from Sigma-Aldrich.

### Wound Healing Scratch Assay

Mvt1-scrambled and ATF5-KD cells were seeded on six-well plates at a concentration of 3 × 10^5^ cells/well and allowed to adhere for 24 h. Cells were then starved with SFM + 1% BSA overnight. The monolayers were mechanically disrupted with a sterile 10 µl tip. Cells were grown in SFM + 1% BSA for another 48 h. Images were taken with an Axio Observer.Z1 (Carl Zeiss, Oberkochen, Germany), equipped with AxioCam MRm Rev.3 camera (Carl Zeiss) at 5× magnification at times 0, 24, and 48 h from scratch introduction, at the same coordinates for each image at all time points. Scratch area was assessed using Zen 2.3 lite software (Carl Zeiss) and presented as % of scratch area at time point 0.

### Transwell Migration Assay

Mvt1 or Met1 cells were seeded in medium supplemented with 1% FBS on the upper chamber of 8 µm hanging cell culture inserts for 24-well plates (Merck Millipore, Billerica, MA, USA) at a concentration of 1.5 × 10^5^ cells/well. Medium supplemented with 10% FBS was added to the lower chamber of the well. Cells were allowed to migrate for 20 h. At the end of the experiment, cells were washed with PBS, fixated with 4% paraformaldehyde, perforated with 100% methanol, and stained with Giemsa solution (Merck Millipore). The cells from the upper side of the membrane (non-migrated cells) were removed. Images of stained migrated cells on the lower side of the membrane were taken with an Axio Observer.Z1 (Carl Zeiss), equipped with AxioCam ICc3 camera (Carl Zeiss) at 20× magnification. Stained area was assessed using ImagePro Plus 6.0 software (Media Cybernetics, Inc., Rockville, MD, USA) by counting the number of stained pixels in the images and presented as % of migrated scrambled cells.

### Images of Cultured Mvt1 Cells

Mvt1-scrambled and ATF5-KD cells were seeded on six-well plates at a concentration of 1.5 × 10^5^ cells/well and allowed to adhere for 24 h. Images were taken with an Axio Observer.Z1, equipped with AxioCam MRm Rev.3 camera at 10× magnification.

### Flow Cytometry

Mvt1-scrambled and ATF5-KD cells were stained with Pacific-Blue-conjugated anti-CD24 antibody (Biolegend, San Diego, CA, USA) at a concentration of 5 × 10^6^ cells/ml of FACS buffer (0.1% BSA in PBS) for 20 min on ice in the dark, and after which, the cells were washed twice and resuspended in PBS containing 1% BSA. Stained cells were analyzed using the CyAn ADP Instrument (Dako-Cytomation, Glostrup, Denmark) and the FlowJo 7.25 analysis software.

### Statistical Analysis

Results are presented as mean ± SEM or mean ± SD. Statistical analyses were performed using GraphPad Prism (Version 5; GraphPad Software Inc.). Student’s *t*-test and one-way and two-way analyses of variance (ANOVA), followed by Bonferroni multiple comparison posttest, were used to determine the statistical significance of differences between groups. *P* < 0.05 was considered statistically significant.

## Results

**Figure 1:**
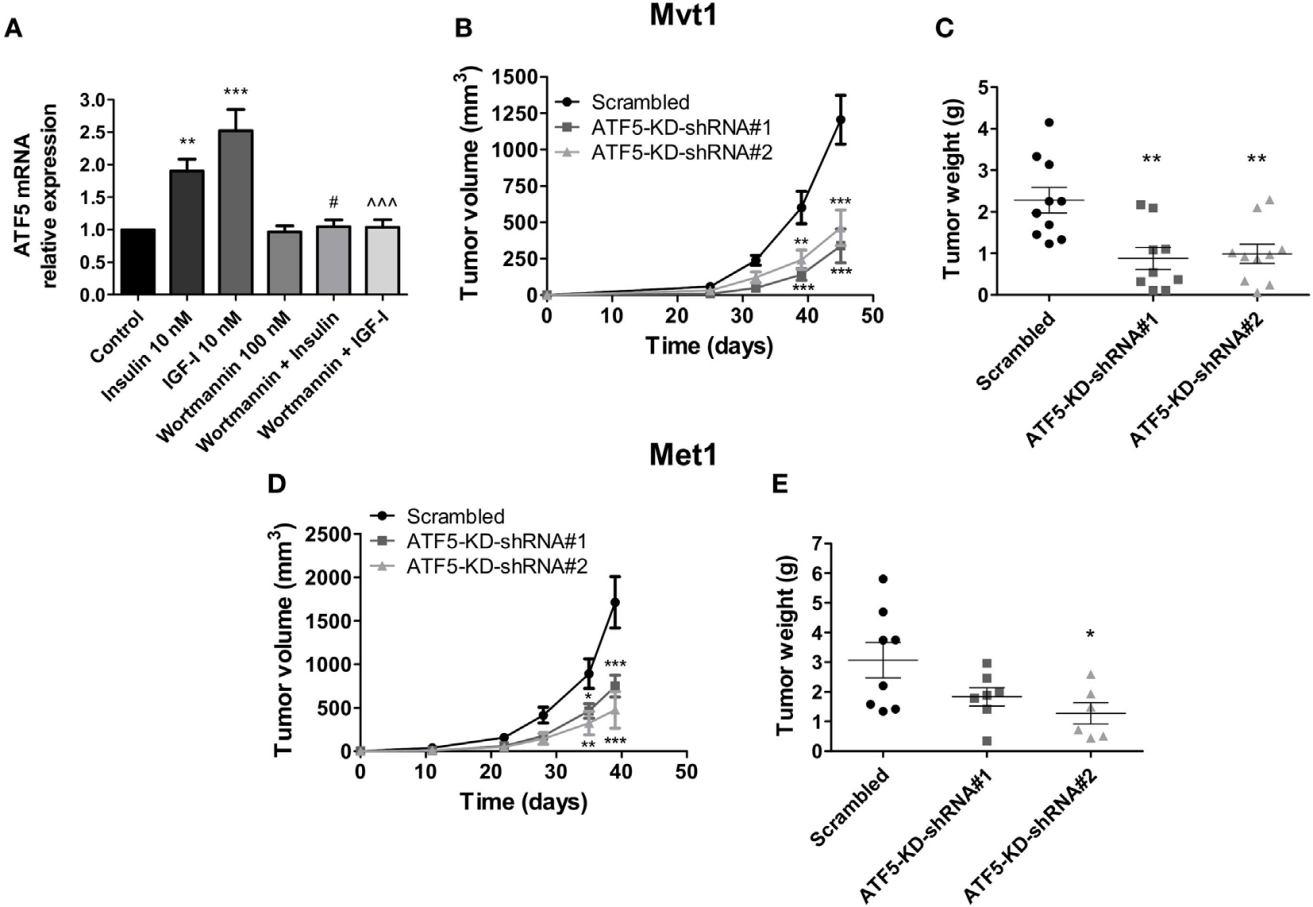
Activating transcription factor-5 (ATF5) knockdown reduces mammary tumor growth. **(A)** Relative mRNA levels of ATF5 were analyzed by real-time PCR in Mvt1 cells pre-incubated in the presence or absence of wortmannin (100 nM) for 30 min before the addition of insulin of insulin-like growth factor-I (IGF-I) (10 nM) for additional 4 h. Results represent mean ± SEM of five independent experiments, *n* = 9; one-way analysis of variance (ANOVA) followed by Bonferroni multiple comparison posttest was used to determine statistical significance; **P < 0.01, ***P < 0.001 vs. control, ^#^*P* < 0.05 vs. insulin, ^∧∧∧^*P* < 0.001 vs. IGF-I, *n* = 9. **(B)** Tumor volume was assessed at the indicated time after the inoculation of Mvt1-scrambled cells or ATF-KD cells, derived from two different short hairpin RNA (shRNA) constructs, shRNA#1 and shRNA#2, into the mammary fat pad of FVB/N female mice (50,000 cells per mice), *n* ≥ 9. **(C)** Tumor weight was measured at sacrifice, *n* ≥ 9. **(D)** Tumor volume was assessed at the indicated time after the inoculation of Met1 scrambled cells or ATF-KD cells into the mammary fat pad of FVB/N female mice (500,000 cells per mice), *n* = 4. **(E)** Tumor weight was measured at sacrifice, *n* = 4. Results are representatives of two independent experiments and presented as mean ± SEM. Student’s *t*-test **(E)**, two-way ANOVA **(B,D)**, and one-way ANOVA **(C)** followed by Bonferroni multiple comparison posttest were used to determine statistical significance; **P < 0.01, ***P < 0.001 vs. scrambled.

First, we measured ATF5 mRNA levels in Mvt1 cells treated with insulin or IGF-I at a concentration of 10 nM each. As shown in Figure 1A, insulin induced the expression of ATF5 mRNA in Mvt1 cells by approximately twofold within 4 h. Similarly, exposure of the cells to IGF-I for 4 h resulted in a significant increase of ~2.5-fold in mRNA levels of ATF5. Pre-incubation of cells with the PI3K inhibitor, wortmannin (100 nM), abrogated both insulin- and IGF-I-induced expressions of ATF5 mRNA, indicating that ATF5 induction is dependent on activation of the PI3K/Akt signaling pathway. These results suggest that ATF5 may play a role in the intracellular signaling mechanisms, which mediate the known effects of insulin and IGF-I on cell proliferation and mammary tumor growth (5, 25). Therefore, we next investigated the effect of ATF5 knockdown in Mvt1 and Met1 cells on tumor growth, cell proliferation, and migration and intracellular signaling pathways.

**Figure 2:**
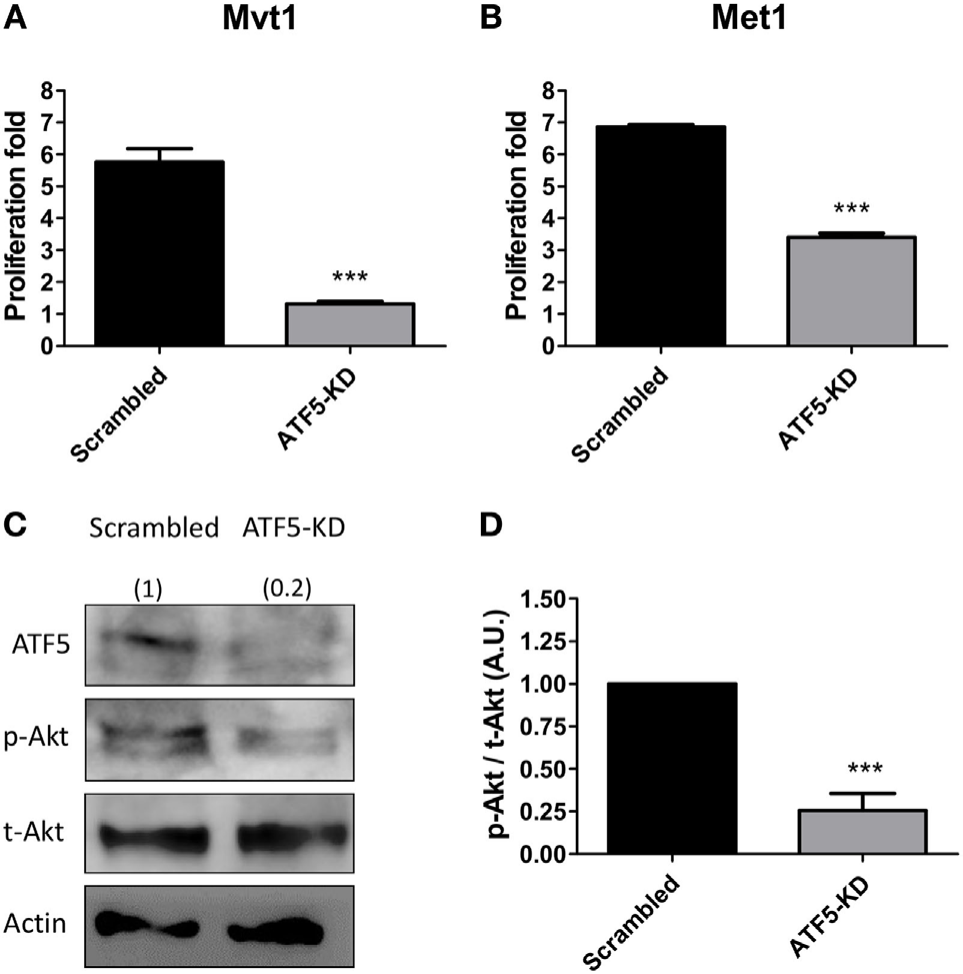
Activating transcription factor-5 (ATF5) knockdown decreases the proliferation rate and Akt activation in mammary tumor cells. Scrambled and knockdown of ATF5 (ATF5-KD) cells, derived from **(A)** Mvt1 cell line or **(B)** Met1 cell line, were grown for 72 h in 96-well plates and subjected to proliferation assay analysis. Proliferation fold was calculated as the ratio between the fluorescent values at the end of the experiment and time 0. Results are representatives of two independent experiments and presented as mean ± SEM, *n* = 4; Student’s *t*-test was used to determine statistical significance; ***P < 0.001. **(C)** Protein was extracted from scrambled and ATF5-KD Mvt1 cells, and Western blot analysis was performed using antibodies directed against ATF5, phospho-Akt (Thr308), total-Akt, and β-actin. **(D)** Densitometry analysis was performed using the ImageQuant software. Results represent mean ± SEM in arbitrary units (A.U.) of four independent experiments, *n* = 4; Student’s *t*-test was used to determine statistical significance; ***P < 0.001.

Introducing two different shRNA constructs (shRNA#1 and #2) against ATF5 to Mvt1 and Met1 cells resulted in a significant decrease of 80% compared to control levels in mRNA levels of ATF5 in both cell lines (data not shown). Protein levels of ATF5 were also decreased in Mvt1 and Met1 cells by 80 and 70%, respectively (Figures 2C and 5C, respectively). Inoculation of Mvt1 cells expressing a control vector (scrambled) or each of the knockdown vectors (ATF5-KD-shRNA#1 or ATF5-KD-shRNA#2) into the mammary fat pad of female FVB/N mice resulted in tumors that were measurable on day 25 after inoculation. Weekly assessments of tumor volume, as shown in Figure 1B, show a significant decrease of more than 60% in the growth rate of ATF5-KD tumors compared to tumors comprising scrambled Mvt1 cells. Figure 1C shows tumor weights at the end of the experiment (day 45 after inoculation of cells), where the tumors derived from both ATF5-KD-shRNA#1 and #2 Mvt1 cells were significantly smaller than scrambled tumors by greater than twofold. Similar changes in tumor volume and weight were obtained with scrambled and ATF5-KD-shRNA#2 cells (hereafter referred to as ATF5-KD) derived from Met1 cells (Figures 1D,E).

In order to understand the possible cellular mechanisms underlying the observed ATF5-KD-mediated reduction in mammary tumor growth rates, we performed various *in vitro* experiments, to assess cell proliferation rate, PI3K/Akt activation level and migratory potential of ATF5-KD cells compared to scrambled cells. Scrambled and ATF5-KD Mvt1 cells as well as Met1 cells were grown for 72 h, and cell proliferation was measured by a commercially available kit as described above. During the time of the experiment, Mvt1-scrambled cells increased in numbers to about six times of the initial seeding cell number, while ATF5-KD cells’ number at the end of the experiment was less than 1.5-fold of the initial number (Figure 2A). Similarly, ATF5-KD Met1 cells showed a reduced cell proliferation rate of about half the proliferation rate that was observed for scrambled Met1 cells (Figure 2B). Analysis of PI3K/Akt signaling pathway by measuring phosphorylated Akt (p-Akt) levels in scrambled and ATF5-KD Mvt1 cells revealed a marked reduction of 75% in Akt activation levels in ATF5-KD cells compared with scrambled cells (Figures 2C,D).

**Figure 3:**
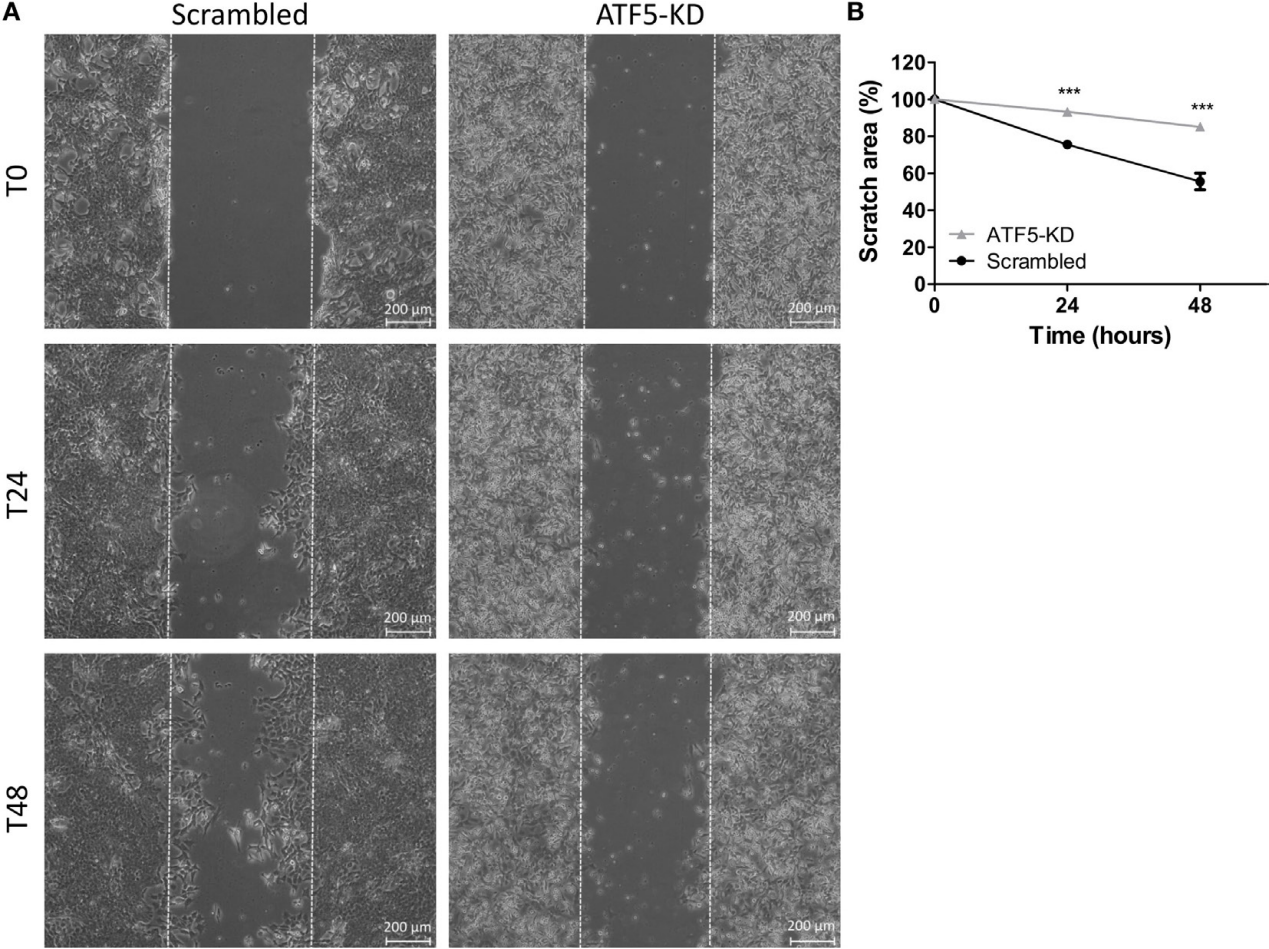
Activating transcription factor-5 (ATF5) knockdown inhibits the wound healing rate of mammary tumor cells. **(A)** Scrambled and knockdown of ATF5 (ATF5-KD) Mvt1 cells were grown in serum-free medium + 1% bovine serum albumin for 48 h after a mechanical disruption of the monolayers. Images were taken at time points 0, 24, and 48 h after performing the scratch at the same coordinates for each image. **(B)** Scratch area is presented as % of scratch area at time point 0. Results are representatives of two independent experiments and presented as mean ± SEM, *n* = 3. Two-way analysis of variance, followed by Bonferroni multiple comparison posttest, was used to determine statistical significance; ***P < 0.001 vs. scrambled.

**Figure 4:**
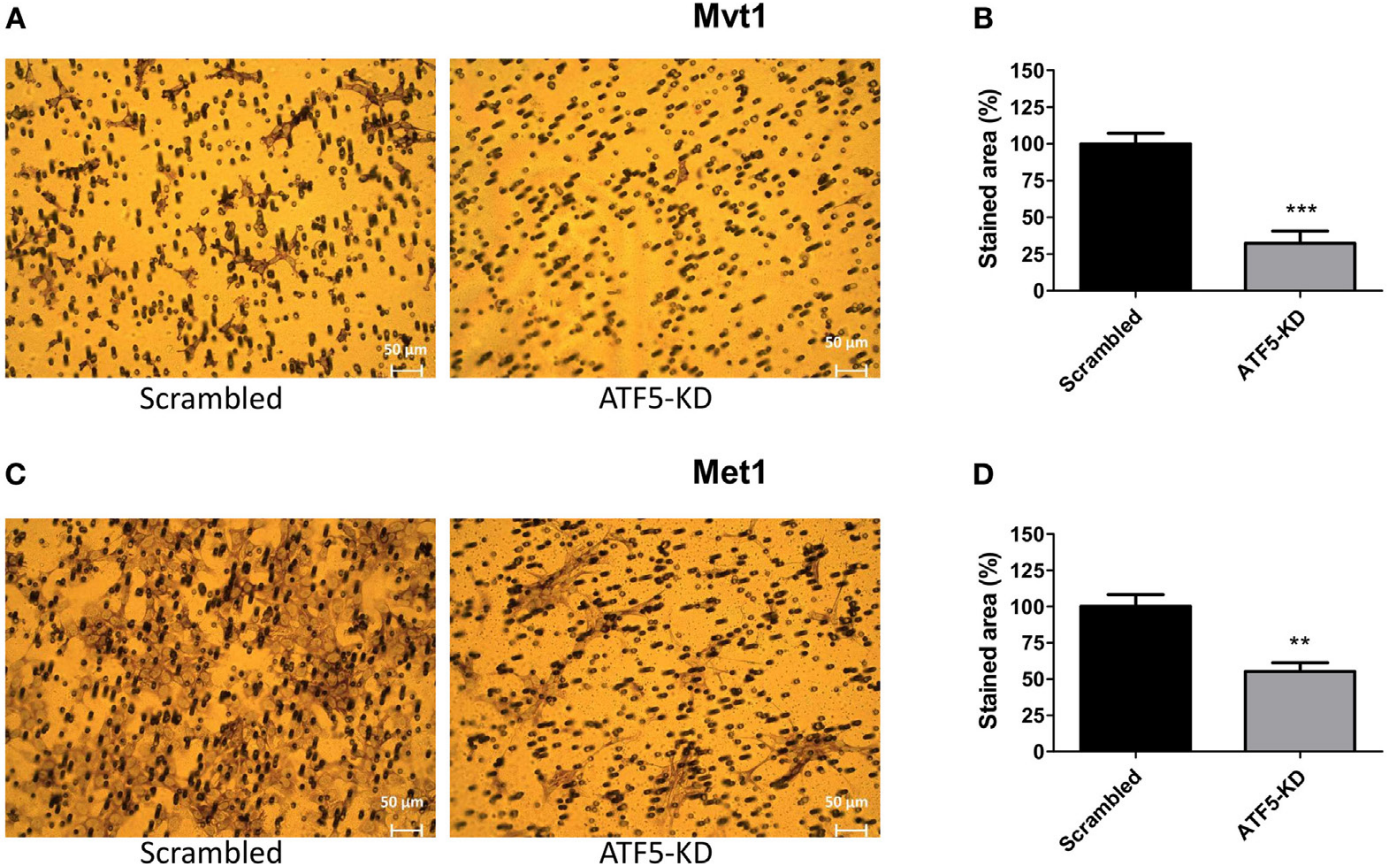
Activating transcription factor-5 (ATF5) knockdown impairs the migratory potential of mammary tumor cells. Scrambled and knockdown of ATF5 (ATF5-KD) cells, derived from **(A)** Mvt1 cell line or **(C)** Met1 cell line, were seeded on the upper chamber of 8 µm hanging cell culture inserts and incubated for 20 h. Cells were stained with Giemsa solution, and images of migrated cells (on the lower side of the membrane) were taken. Stained area was assessed by counting the number of stained pixels in the images and presented as % of migrated scrambled cells derived from **(B)** Mvt1 cell line or **(D)** Met1 cell line. Results are representatives of two independent experiments and presented as mean ± SEM, *n* = 4; Student’s *t*-test was used to determine statistical significance; **P < 0.01, ***P < 0.001.

To assess the migratory potential of ATF5-KD cells compared to scrambled cells, we used the following two common assays: wound healing scratch assay and transwell migration assay. Figure 3 depicts the results of the scratch assay obtained for Mvt1 cells. The scratch area of both scrambled and ATF5-KD cells at time 0 (immediately after performing the scratch) is 100%. While scrambled cells closed 25% of the scratch wound after 24 h and almost 50% of the scratch area after 48 h, ATF5-KD cells were able to cover only 7 and 15% of the scratch wound at time 24 and 48 h, respectively. Transwell migration assay also revealed an impaired migratory potential of ATF5-KD cells, since the cell-stained area of the ATF5-KD Mvt1 cells was only 33% of the stained area obtained with scrambled Mvt1 cells (Figures 4A,B). Similarly, the cell-stained area of the ATF5-KD Met1 cells was 50% of the stained area obtained with scrambled Met1 cells (Figures 4C,D).

**Figure 5:**
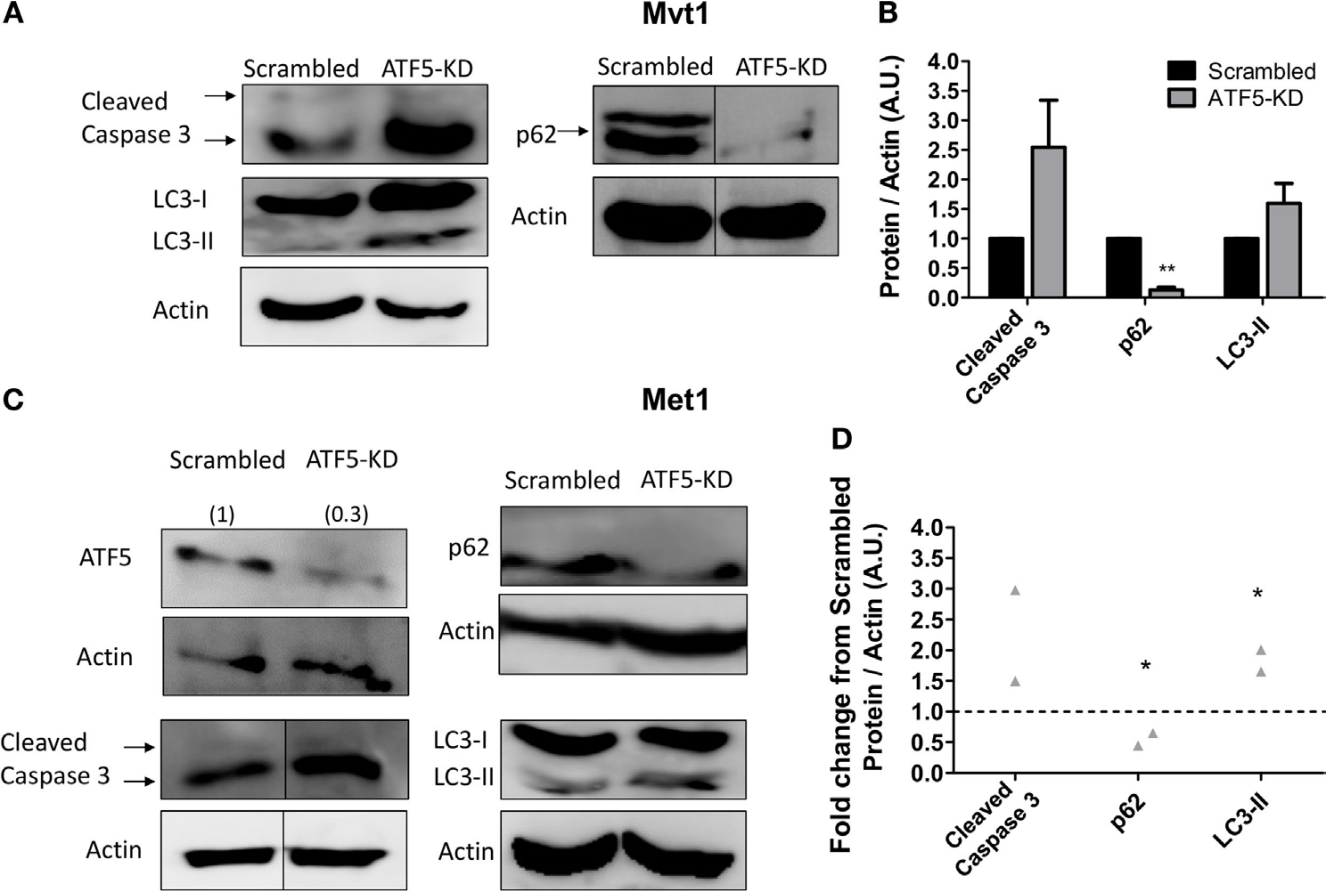
Activating transcription factor-5 (ATF5) knockdown induces autophagy in mammary tumor cells. Scrambled and knockdown of ATF5 (ATF5-KD) cells, derived from **(A)** Mvt1 cell line or **(C)** Met1 cell line, were grown in serum-free medium + 1% bovine serum albumin for at least 72 h. Protein was extracted, and Western blot analysis was performed using antibodies directed against ATF5, caspase 3, light chain 3 (LC3), p62, and β-actin. Note, for p62 in Mvt1 cells and for cleaved caspase 3 in Met1 cells, and the corresponding actin results, bands were grouped from different parts of the same blot. The images of the uncropped blots are shown in Figures S1 and S2 in Supplementary Material. **(B,D)** Densitometry analysis was performed using the ImageQuant software. **(B)** Results represent mean ± SEM in arbitrary units (A.U.) of at least three independent experiments, *n* ≥ 3; **(D)** Results represent the fold change of protein expression normalized to actin levels in ATF5-KD cells relative to scrambled cells of two independent experiments, *n* = 2; Student’s *t*-test was used to determine statistical significance; *P < 0.05, **P < 0.01.

Since ATF5 in known to regulate the expression of genes related to apoptosis (16) and autophagy (19), we decided to investigate the effect of ATF5-KD on several markers of apoptosis and autophagy in Mvt1 cells. As shown in Figure 5, ATF5-KD showed a trend toward increased cleaved caspase 3 levels (which did not reach the threshold of statistical significance) in Mvt1 cells (Figures 5A,B) and in Met1 cells (Figures 5C,D). The levels of LC3B-II were also elevated as a result of ATF5 knockdown, and though this increase was only a trend for Mvt1 cells (Figures 5A,B), it was statistically significant in Met1 cells (Figures 5C,D). In addition, levels of p62 were markedly decreased in ATF5-KD Mvt1 cells (Figures 5A,B) and Met1 cells (Figures 5C,D).

**Figure 6:**
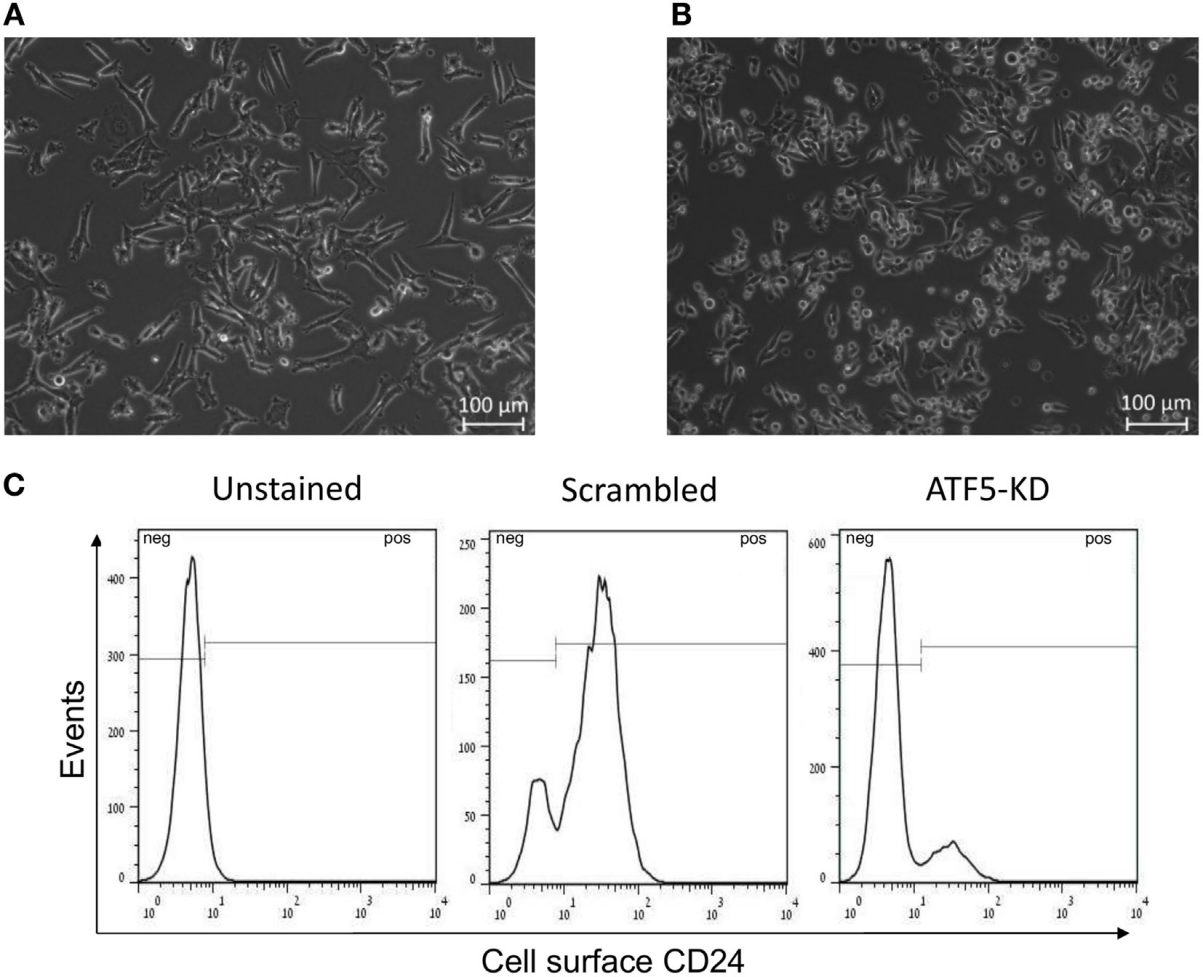
Activating transcription factor-5 (ATF5) knockdown leads to a shift in Mvt1 cells sub-populations toward CD24-negative cells. **(A)** A representative image of Mvt1-scrambled cells. **(B)** A representative image of Mvt1-knockdown of ATF5 (ATF5-KD) cells. **(C)** Scrambled and ATF5-KD Mvt1 cells were stained with Pacific-Blue-conjugated anti-CD24 antibody. Results are representatives of two independent experiments, showing FACS histograms of cell surface expression of CD24 in unstained Mvt1 cells (negative control) as well as stained scrambled and ATF5-KD Mvt1 cells.

Importantly, it appears that ATF5-KD in Mvt1 cells change the morphology of the cells in culture. While scrambled Mvt1 cells were characterized by an elongated and spindle-like cytoplasm (Figure 6A), ATF5-KD culture was enriched with rounded cells (Figure 6B). These changes in morphology are similar to the different phenotypes that we have previously identified for CD24-positive and CD24-negative sub-populations of Mvt1 cells, which were characterized by the presence or absence of CD24 on their cell surface (13, 26). These results led us to examine scrambled and ATF5-KD Mvt1 cells for their cell surface expression of CD24. Interestingly, ATF5-KD resulted in a prominent shift in the sub-populations comprising Mvt1 cells. While scrambled Mvt1 cells were mainly CD24-positive cells, ATF5-KD cells were mostly CD24-negative cells (Figure 6C; Table 1).

**Table 1:** %CD24-positive cells from total population.

Scrambled	Knockdown of activating transcription factor-5
83.0 ± 0.8	32.5 ± 14.4*

Data represent the mean ± SEM, n = 3; Student’s t-test was used to determine statistical significance, *P < 0.05.

## Discussion

The results of this study suggest that ATF5 plays a major role in promoting tumor growth of Mvt1 and Met1 mammary tumor cells. The importance of ATF5 in mediating this effect was evident by the reduced tumor volume and weight derived from cells in which ATF5 expression was knocked down, as well as by the decrease in proliferation, migration, and activation of pro-survival signaling pathways, such as the PI3K/Akt pathway, in ATF5-knockdown cells *in vitro*.

Up until a decade ago, ATF5 had not been extensively studied outside the brain. Previous reports have shown that ATF5 is expressed in neuroprogenitor cells and that, when ATF5 is constitutively expressed in these cells, it prevents them from differentiating and allows their continuous proliferation (27). In 2007, Monaco et al. showed that ATF5 is widely expressed in several carcinomas, including glioblastomas and breast cancer cell lines, and that interfering with its function selectively kills neoplastic but not non-transformed cells (22). Since then, ATF5 became a gene of interest and reports of its expression and its role in promoting the survival of various stress-induced cancer cells have been published (16–18). The mechanism proposed for the cell-specific pro-survival function of ATF5 is the induction of anti-apoptotic factor BCL-2 expression by ATF5. It was suggested the BCL-2 expression is regulated by ATF5 in cancer cells but not in non-transformed cells (16). In the present study, we also show that ATF-KD mammary tumor cells are characterized by a reduced proliferation and tumor growth. However, apoptotic regulation may not be the principal mechanism underlying this effect, since the elevation in cleaved caspase 3 levels did not reach statistical significance. In contrast, the marked reduction in the activation of PI3K/Akt signaling pathway, as evidenced by decreased p-Akt levels, may indicate a reduced proliferation rate of ATF5-KD mammary tumor cells. Importantly, previous studies, investigating the role of ATF5 in survival of breast cancer cell lines, have focused so far on experiments performed *in vitro*, such as proliferation, migration, and apoptosis assays, and intracellular mechanisms by which ATF5 may exert its effects (16, 22, 28, 29). Therefore, to our knowledge, we show here for the first time that ATF5 downregulation leads to a decreased mammary tumor growth *in vivo*.

In addition to the reduction in proliferation rate, ATF5-KD cells also showed an increased autophagy flux in Mvt1 and Met1 cells, as assessed by changes in protein levels of the known autophagy markers, LC3-II (increased) and p62 (decreased). Similar results were reported in transformed murine myeloid cells by Sheng et al., who suggested that ATF5 may regulate autophagy through mammalian target of rapamycin, a master negative regulator of autophagy (30), which was revealed as a direct target gene of ATF5 transcription factor (19, 30). Although, autophagy is often considered as a pro-survival mechanism protecting the cell against dwindling nutrient resources (31), studies in the beclin 1 autophagy gene knockout mice suggest that autophagy may also have a tumor suppressor function (32). Thus, it is possible that an increased autophagy and a reduced proliferation rate of ATF5-KD Mvt1 and Met1 cells lead to the reduced tumor growth shown in the present study.

The mitogenic effects of insulin and IGF-I are well known and stand at the basis of the association between T2D and an increased risk of breast cancer incidence and mortality (4–6). As we have recently shown, insulin or IGF-I stimulation of Mvt1 cells leads to the upregulation of several mRNA transcripts, associated with cell proliferation and migration, such as cyclin D1 and the transcription factor ETS2, as well as to the downregulation of the transcriptional repressor, HBP1 (12). Here, we show for the first time that ATF5 is also upregulated after exposure to either insulin or IGF-I and suggest that the ATF5 can also mediate the proliferative effects of the two ligands, which can subsequently lead to an increased mammary tumor growth. Interestingly, the presence of the PI3K inhibitor, wortmannin, abrogated the insulin- and IGF-I-induced upregulation of ATF5. This suggests that the ATF5 expression is dependent on PI3K/Akt activation upon stimulation with insulin and IGF-I. Moreover, we show that activation of Akt is reduced in ATF5-KD cells, which suggests that PI3K/Akt activation is regulated by ATF5. Taken together, we suggest a positive feedback mechanism between PI3K/Akt signaling and ATF5, where PI3K/Akt activation by insulin or IGF-I promotes ATF5 expression in breast cancer cells, and ATF5 further promotes the activation of PI3K/Akt pathway, thereby emphasizing the importance of targeting ATF5 in anticancer therapy, particularly, in diabetic patients with breast cancer.

Further to the effects of ATF5 knockdown on mammary tumor cell proliferation, we show here that migration is also impaired in ATF5-KD Mvt1 and Met1 cells. Very few studies have reported the association between ATF5 expression and cell motility. Among them, Nukuda et al. performed an invasion assay in highly invasive cell lines, such as human HT-1080 fibrosarcoma, A549 lung cancer, and MDA-MB-231 breast cancer, and revealed that ATF5 knockdown reduces the invasiveness of these cells (29). Moreover, the authors showed that ATF5 expression was markedly increased in the invasive MDA-MB-231 cells than in the less invasive breast cancer cell line, MCF-7. Interestingly, the authors also reported that ATF5 knockdown in HT-1080 and MDA-MB-231 cells, which are normally characterized by spindle-like morphology associated with high invasion rate, resulted in a morphology change toward a rounded shape and decreased invasiveness. The mechanism proposed by the authors to the shift in morphology was ATF5-mediated expression of integrin-β1 and integrin-α2 (which was reduced in ATF5-KD lung cancer cells). Here, we report similar changes in morphology in ATF5-KD Mvt1 cells. Furthermore, we show a shift in the sub-populations comprising Mvt1 cells from the aggressive CD24-positive cells toward less aggressive CD24-negative cells. Importantly, CD24 was reported to induce the recruitment of integrin-β1 to lipid rafts and activate integrins-α3β1 and α4β1 in breast cancer cells, thereby leading to an increased motility of the cells (33, 34). Moreover, depletion of integrin-β1 in basal epithelial cells of the mammary gland *in vivo* resulted in a decreased surface expression of CD24 in these cells (35). Taking into consideration that CD24-positive Mvt1 cells are also characterized by an increased mRNA expression of ATF5 (13), we suggest that reduction in integrin-β1 expression and/or activation caused by ATF5 knockdown in Mvt1 cells may stand at the basis of the observed changes in morphology and the shift from CD24-positive cells to CD24-negative Mvt1 cells. As CD24 expression is often associated with CD44 expression in breast cancer cells (36), we analyzed CD44 cell surface expression in Mvt1-derived scrambled and ATF5-KD cells. However, both control and ATF5-silenced cells showed a positive CD44 expression (data not shown), indicating that in Mvt1 cells, ATF5-KD does not affect cell surface expression of CD44. It should also be noted that as Mvt1 cell line naturally comprises two sub-populations of both CD24-positive and CD24-negative cells, the regulation mechanism of CD24 expression in these cells may be different than other mammary tumor cells that are characterized as “pure” CD24-expressing cells, such as Met1 cell line, in which ATF5-KD did not affect the cell surface expression of CD24 (data not shown). Therefore, we suggest that ATF5 may be involved in the type of regulation mechanism of CD24 cell surface expression as manifested in Mvt1 cell line.

Taken together, the results of the present study emphasize the role of ATF5 in enhancing mammary tumor cells proliferation, migration, and overall aggressiveness, thereby promoting mammary tumor growth. Targeting ATF5 in anticancer therapy may be particularly attractive because of its differential role in cancer cells than in non-transformed cells, thus allowing specificity of the treatment. In addition, we have previously reported that CD24 cell surface expression in Mvt1 cells may serve as a biomarker for sensitivity to anti-IGF-I-receptor therapy (26). The results of the present study suggest that breast cancer patients with CD24 cell surface expressing cancer cells may also benefit from anti-ATF5 therapy. Thus, combining anti-IGF-I receptor and anti-ATF5 therapies may show good efficiency in attenuating aggressive CD24-positive tumor growth.

## Ethics Statement

Ethics number: IL-019-02-2017. Technion Committee for Care and Use of Laboratory Animals.

## Author Contributions

SB-S developed the ideas, conducted the experiments, analyzed the data, drafted and revised the manuscript; RRa conducted the experiments; RRo developed the ideas and revised the manuscript; EI conducted the experiments; ZS-O provided the administrative support; and DL supervised the study, developed the ideas, and revised the manuscript. All authors read and approved the final manuscript.

## Conflict of Interest Statement

The authors declare that the research was conducted in the absence of any commercial or financial relationships that could be construed as a potential conflict of interest.
