# Supplementary figures and images for: Activating Transcription Factor-5 Knockdown Reduces Aggressiveness of Mammary Tumor Cells and Attenuates Mammary Tumor Growth
Source: Front Endocrinol (Lausanne). 2017 Jul 21;8:173. doi: 10.3389/fendo.2017.00173 (PMC5519529; doi:10.3389/fendo.2017.00173)

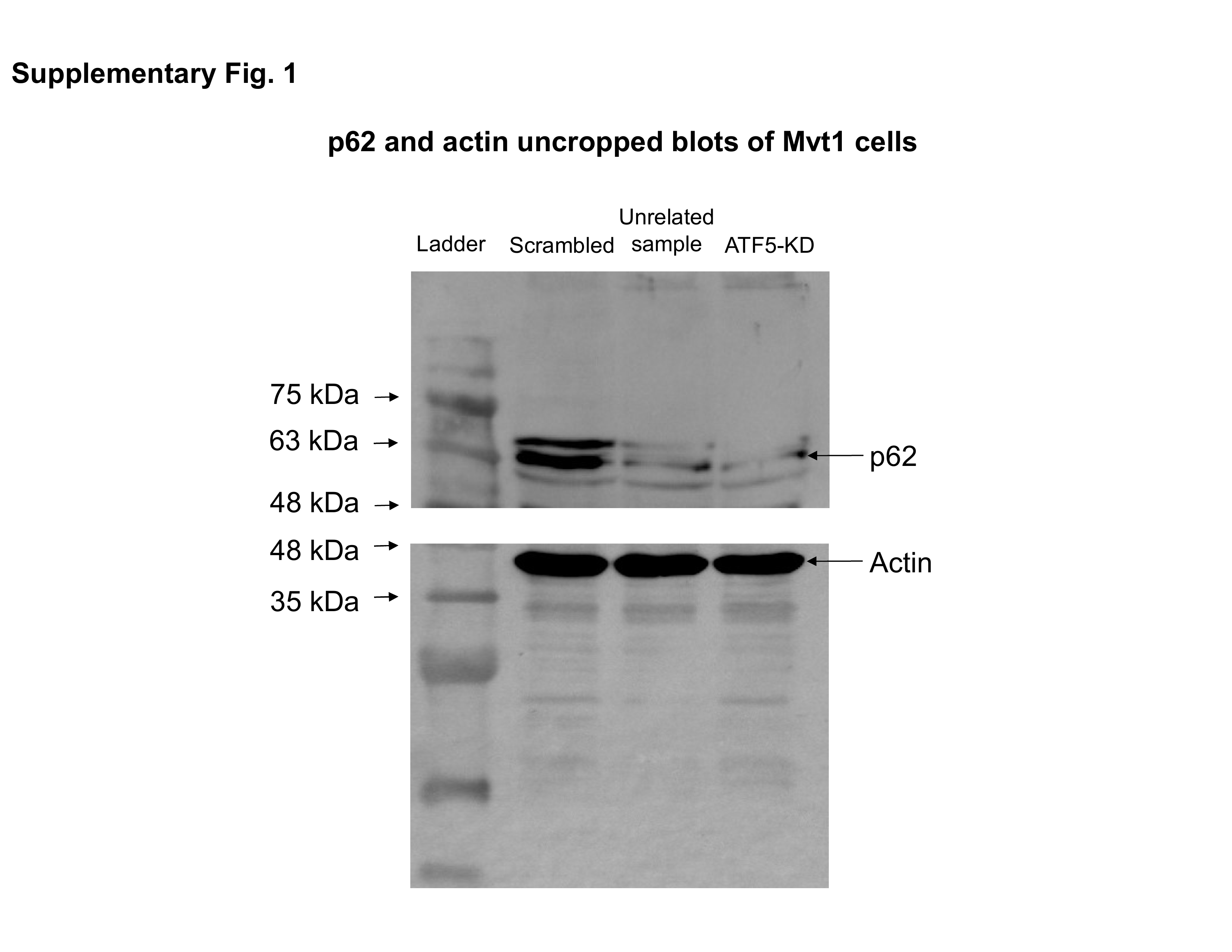

Supplement: Supplementary file 1 [file image_1.tif]

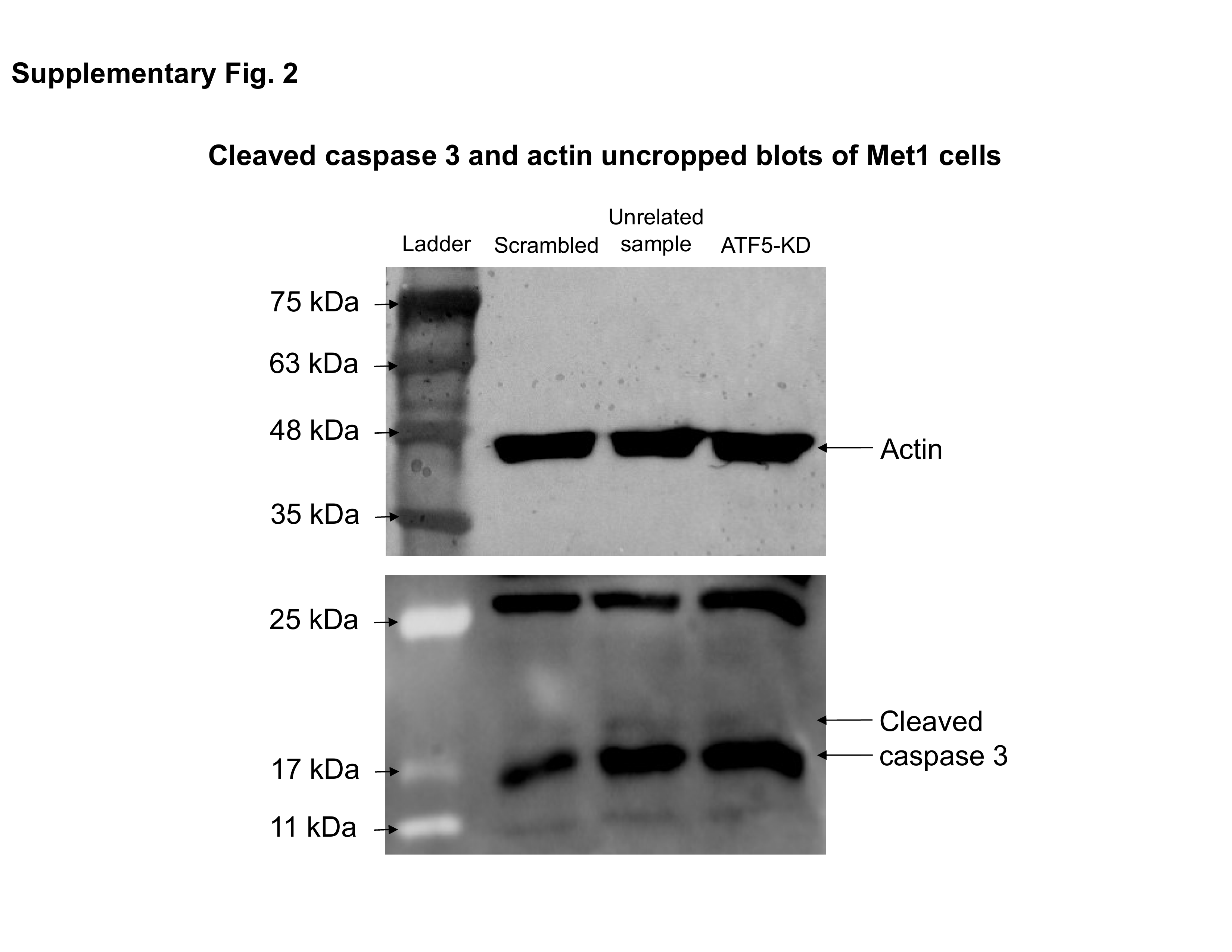

Supplement: Supplementary file 2 [file image_2.tif]
